# The association of vertical and horizontal workplace social capital with employees’ job satisfaction, exhaustion and sleep disturbances: a prospective study

**DOI:** 10.1007/s00420-019-01432-5

**Published:** 2019-04-09

**Authors:** Elisabeth Framke, Ole Henning Sørensen, Jacob Pedersen, Thomas Clausen, Vilhelm Borg, Reiner Rugulies

**Affiliations:** 1 National Research Centre for the Working Environment, Lersø Parkallé 105, 2100 Copenhagen, Denmark; 2 Department of Public Health, University of Copenhagen, Øster Farimagsgade 5, 1014 Copenhagen, Denmark; 3 Department of Psychology, University of Copenhagen, Øster Farimagsgade 2A, 1353 Copenhagen, Denmark

**Keywords:** Employee health, Employee well-being, Social capital, Vertical, Horizontal, Aggregated measures

## Abstract

**Purpose:**

Workplace social capital (WSC) may be beneficial for employees’ health and well-being; however, most studies have analyzed WSC on the individual and not the workplace level. We test whether higher compared to lower levels of vertical WSC (WSC between employees and superiors) and horizontal WSC (WSC between employees), measured at the workplace level, is prospectively associated with higher levels of employees’ well-being.

**Methods:**

Using data from an intervention study, we analyzed associations between workplace aggregated vertical and horizontal WSC at baseline with job satisfaction, exhaustion and sleep disturbances at 24-months follow-up. The sample included 606 municipal pre-school employees (71 workplaces). We adjusted for individual and workplace characteristics, baseline scores of outcomes, intervention status, and the interaction of exposure with intervention status. We used the Genmod procedure in SAS with a repeated statement to account for correlation of individuals within workplaces. We repeated analyses using individual-level WSC measurements.

**Results:**

Higher levels of vertical and horizontal WSC at baseline predicted a higher level of job satisfaction (0.20, *p* = 0.01 and 0.24, *p* = 0.01, respectively) and a lower level of exhaustion (− 0.33, *p* = 0.04 and − 0.43, *p* = 0.04) at follow-up in the most adjusted model. Analyses with individual-level measures yielded similar results and further showed an association of a higher level of horizontal WSC with a lower level of sleep disturbances.

**Conclusions:**

Higher levels of vertical and horizontal WSC were prospectively associated with better well-being of employees in municipal pre-schools. Workplaces may thus consider focusing on improving WSC as a means for ensuring or improving employees’ well-being.

**Electronic supplementary material:**

The online version of this article (10.1007/s00420-019-01432-5) contains supplementary material, which is available to authorized users.

## Introduction

Social capital has been conceptualized as resources in social relations among individuals in social units (Berkman and Kawachi 2000). The concept of workplace social capital (WSC) refers to actual and potential resources in cooperative relations between employees at the workplace, e.g., in work teams, and in relations between employees and their superiors (Meng et al. 2018; Oksanen et al. 2010). A high level of WSC is characterized for example by a workplace climate of social support, mutual trust, and constructive cooperative relations between employees and between employees and their superiors (Meng et al. 2018). A high level of WSC may both enhance the ability for employees to deal with their job demands and contribute to sustaining employees’ psychological well-being (Bakker and Demerouti 2016).

An increasing number of studies have reported that higher levels of WSC are associated with higher levels of health and well-being in employees (Kawachi et al. 2013). However, most studies are based on cross-sectional designs. Exceptions are research reports from the Finnish Public Sector study (Oksanen et al. 2010, 2011) and recent studies of WSC as a predictor of long-term sickness absence (Hansen et al. 2018; Rugulies et al. 2016; Torok et al. 2018), job satisfaction and work engagement (Stromgren et al. 2016), and mental distress (Tsuboya et al. 2015).

With a few exceptions (Hansen et al. 2018; Kouvonen et al. 2008; Torok et al. 2018), most studies investigating the association between WSC and health and well-being analyzed WSC on the individual level. This is problematic, as WSC is a group phenomenon and therefore ideally should be analyzed as a group-level construct. Further, in most studies on WSC and health and well-being, WSC has been analyzed as a global measure, i.e., as a unidimensional construct without considering potential sub-dimensions, such as vertical (linking) or horizontal (bonding or bridging) WSC (Meng et al. 2018; Szreter and Woolcock 2004).

In this study, we test the hypothesis that higher compared to lower levels of vertical and horizontal WSC, measured at the workplace level, are prospectively associated with higher levels of employees’ well-being. Vertical WSC refers to cooperative relations between employees and their superiors, while horizontal WSC refers to cooperative relations between colleagues. We use three measures of employees’ well-being; job satisfaction, exhaustion and sleep disturbances. In supplementary analyses, we examined if the observed associations could be reproduced in analyses where WSC was operationalized as an individual-level construct and not a group-level construct.

## Methods

### Study design and population

We analyzed the prospective association between vertical and horizontal WSC at baseline and job satisfaction, exhaustion and sleep disturbances at 24 months of follow-up. We used data from the Pioneer intervention study (Framke et al. 2016, 2018), a cluster-randomized participatory organization-level workplace intervention that aimed to improve well-being and reduce sickness absence by focusing on core job tasks. In September 2011 and September 2013, all employees within 78 municipal pre-schools received a questionnaire on WSC and well-being. Of the 78 workplaces, 7 were lost to follow-up. Therefore, this study is based on data from employees within 71 workplaces. Employees belonged to the following job groups: pedagogical leaders, nursery nurses, nursery nurse assistants, and other job groups. Other job groups were primarily kitchen and cleaning staff and school caretakers.

**Fig. 1:**
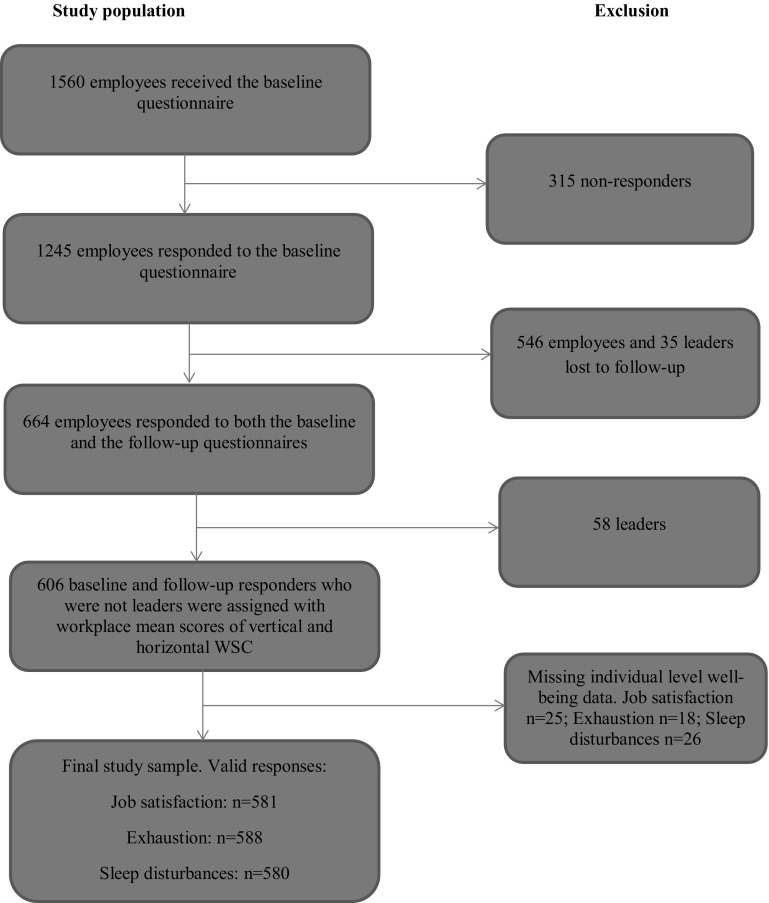
Flowchart of the final study population

Figure 1 shows the flowchart toward the final study population. In total, 1560 employees received the baseline questionnaire, of which 1245 responded (79.8%). Of the 1245 baseline responders, 664 also responded to the follow-up questionnaire 24 months later. We excluded 58 pedagogical leaders, because their WSC may differ from the WSC of the employees, yielding a sample of 606 employees from 71 workplaces. Finally, we excluded employees with missing individual-level data on job satisfaction, exhaustion and sleep disturbances, yielding a final study sample of *n* = 581, *n* = 588 and *n* = 580 for the analyses on job satisfaction, exhaustion and sleep disturbances, respectively.

According to Danish law, research studies that use solely questionnaire and register data do not need approval from the National Committee on Health Research Ethics (Den Nationale Videnskabetiske Komité).

### Measurement of vertical and horizontal WSC

We measured WSC with self-administered questionnaires in September 2011. Of the nine WSC items, five were derived from the Danish Work Environment Cohort Study (DWECS) from the year 2010 (Det Nationale Forskningscenter for Arbejdsmiljø 2011), one was derived from DWECS from the year 2005 (Feveile et al. 2007), one was derived and slightly modified from a questionnaire on relational coordination (Hoffer et al. 2008), and two items were developed for the purpose of this study. A factor analysis (rotation method: varimax) showed two distinct factors with eigenvalues of 4.14 and 1.78, respectively. We named these factors “vertical WSC” (i.e., social capital in the relationship between employees and their superiors, five items) and “horizontal WSC” (i.e., social capital in the relationship between employees, four items) in accordance with theoretical considerations about different types of social capital in the literature (Oksanen et al. 2010). All rotated factor loadings were > 0.70 for vertical WSC and > 0.65 for horizontal WSC. Cronbach’s alpha was 0.87 and 0.80 for vertical and horizontal WSC, respectively.

A sample item for vertical WSC was “We have confidence in the management”, a sample item for horizontal WSC was “We help each other in achieving the best possible results”. Online Appendix 1 shows the full list of items and their response categories. Respondents were included if they responded to at least three of the five items of the vertical component of WSC and to at least two of the four items of the horizontal component of WSC. Response categories ranged from ‘To a very small extent’ (1) to ‘To a very large extent’ (5).

We calculated mean scores of vertical and horizontal WSC for each of the 71 workplaces. Then, we assigned the workplace-specific mean scores to all individuals working at the same workplace. Intra-class correlations were 0.35 and 0.14 for vertical and horizontal WSC, respectively, indicating that a considerable proportion of the variation in vertical and horizontal WSC was explained by the workplace and that the aggregation of the individual-level scores to the workplace level was justified.

### Measurement of job satisfaction, exhaustion and sleep disturbances

We assessed employees’ well-being with three measures: job satisfaction, exhaustion and sleep disturbances. All three measures have been frequently used in studies on working conditions and employees’ well-being (Bond and Bunce 2001; Faragher et al. 2005; Lindeberg et al. 2011; Bültmann et al. 2013; Linton et al. 2015). We measured the three outcomes with self-administered questionnaires at baseline and at 24 months of follow-up. Job satisfaction, measured with the item ‘Regarding your work in general. How satisfied are you with your job as a whole, everything taken into consideration?’, was rated on a four-point scale (very satisfied, satisfied, dissatisfied, very dissatisfied) (Pejtersen et al. 2010). We measured exhaustion (‘Within the past 2 weeks, how much of the time have you felt lacking in energy and strength?’) and sleep disturbances (‘Within the past 2 weeks, how much of the time have you had trouble sleeping at night?’) with one item each, derived from the Major Depression Inventory (Bech et al. 2001). There were six response categories (all of the time, most of the time, slightly more than half of the time, slightly less than half of the time, some of the time, at no time). Higher scores indicate a higher level of job satisfaction, a higher level of exhaustion and a higher level of sleep disturbances.

### Measurement of covariates

Age, sex, job group, workplace size, workplace type, baseline scores of the three outcomes, intervention status and the interaction term intervention status × predictor variable were included as covariates. Age (continuous), sex, job group (nursery nurses, nursery nurse assistants, other job groups), workplace size (number of employees per workplace), workplace type (integrated, day care, kindergarten) and intervention status (intervention vs. control group) were register based.

### Statistical analysis

All analyses were conducted using SAS 9.4.

We tested baseline differences between the study population and the population lost to follow-up with Chi square test and two sample *t* test.

Using the Genmod procedure we estimated the association of workplace aggregated measures of vertical and horizontal WSC at baseline with level of job satisfaction, exhaustion and sleep disturbances at 2 years of follow-up, while taking into account that employees were nested within workplaces. In supplementary analyses, we used individual-level measures of vertical and horizontal WSC instead of workplace aggregated measures of WSC.

We calculated estimates adjusted for sex and age, and further adjusted for job group, workplace type, workplace size and baseline scores of the outcomes. Because the data set was derived from an intervention study, we further adjusted for intervention status and for the interaction term intervention status × predictor variable.

## Results

### Baseline characteristics of the study population

Table 1 shows that the mean age of the study population was 43 years and that 87% were women. Of the total study population, 58% worked as nursery nurses, 32% worked as nursery nurse assistants and 10% worked in other job groups (primarily kitchen staff and school caretakers). Mean workplace size was about 23 employees per workplace. With regard to workplace type, the majority worked in integrated institutions (78%), with the remaining employees working in day care institutions (19%) and kindergartens (4%).

**Table 1:** Baseline characteristics of the study population (*n* = 606) and the population lost to follow-up (*n* = 546)

	Study population				Population lost to follow-up				Chi^2^ (*p*)	*t* (*p*)
	*N*	%	Mean	SD	*N*	%	Mean	SD		
Age			43.19	10.26			39.41	11.75		5.79 (< 0.01)
Sex										
Women	529	87.29			447	81.87			6.53 (0.01)	
Men	77	12.71			99	18.13				
Job group										
Nursery nurses	354	58.42			289	52.93			3.86 (0.14)	
Nursery nurse assistants	194	32.01			192	35.16				
Other job groups	58	9.57			65	11.90				
Workplace size			22.81	8.92			24.57	9.69		− 3.20 (< 0.01)
Workplace type										
Integrated	470	77.56			441	80.77			2.16 (0.34)	
Day care	114	18.81			91	16.67				
Kindergarten	22	3.63			14	2.56				
Workplace social capital										
Vertical	606		3.81	0.47	546		3.69	0.49		4.37 (< 0.01)
Horizontal	606		3.95	0.30	546		3.82	0.35		6.73 (< 0.01)
Well-being										
Job satisfaction	594		3.10	0.64	528		3.08	0.62		0.56 (0.58)
Exhaustion	599		2.85	1.17	536		3.03	1.17		− 2.57 (0.01)
Sleep disturbances	594		2.14	1.33	528		2.16	1.36		− 0.31 (0.76)

### Comparison of the study population with the population lost to follow-up

Table 1 further shows that younger employees and men were more likely to be lost to follow-up. Further, individuals working at workplaces with a high number of employees were more likely to be lost to follow-up. Job group and workplace type were not related to attrition. A higher level of vertical and horizontal WSC was associated with a lower risk of loss to follow-up. Regarding baseline values of the three outcome variables, individuals with a high level of exhaustion were more likely to be lost to follow-up than individuals with a low level of exhaustion. Level of job satisfaction and level of sleep disturbances were not related to risk of loss to follow-up.

### Association of vertical and horizontal WSC with job satisfaction, exhaustion and sleep disturbances

Table 2 shows the associations between the scores of the predictor variables at baseline and the three outcomes after 2 years of follow-up. Higher levels of vertical and horizontal WSC were associated with a higher level of job satisfaction and a lower level of exhaustion at follow-up in all models. In the most adjusted model, estimates were 0.20, *p* = 0.01 (vertical WSC) and 0.24, *p* = 0.01 (horizontal WSC) for job satisfaction and − 0.33, *p* = 0.04 (vertical WSC) and − 0.43, *p* = 0.04 (horizontal WSC) for exhaustion at follow-up. Higher levels of vertical and horizontal WSC were also associated with higher levels of sleep disturbances in some of the models but not in the most adjusted model.

**Table 2:** Associations of workplace aggregated vertical and horizontal workplace social capital at baseline with job satisfaction, exhaustion and sleep disturbances 2 years later

	Job satisfaction			Exhaustion			Sleep disturbances		
	Est	SE	*p*	Est	SE	*p*	Est	SE	*p*
Model 1									
Vertical social capital	0.27	0.06	< 0.01	− 0.49	0.14	< 0.01	− 0.34	0.16	0.03
Horizontal social capital	0.37	0.09	< 0.01	− 0.72	0.18	< 0.01	− 0.75	0.17	< 0.01
Model 2									
Vertical social capital	0.22	0.07	< 0.01	− 0.32	0.15	0.03	− 0.10	0.15	0.50
Horizontal social capital	0.28	0.09	< 0.01	− 0.44	0.19	0.02	− 0.42	0.19	0.02
Model 3									
Vertical social capital	0.21	0.07	< 0.01	− 0.32	0.16	0.04	− 0.07	0.15	0.63
Horizontal social capital	0.25	0.09	< 0.01	− 0.44	0.20	0.03	− 0.38	0.19	0.05
Model 4									
Vertical social capital	0.20	0.08	0.01	− 0.33	0.16	0.04	− 0.05	0.16	0.75
Horizontal social capital	0.24	0.09	0.01	− 0.43	0.21	0.04	− 0.35	0.20	0.08

Estimate (Est) and standard error (SE) for the association of the baseline score in the predictor variable (vertical and horizontal workplace social capital) with outcomes (job satisfaction, exhaustion, sleep disturbances) 2 years later. Workplace identification number is included in a repeated statement

Model 1: adjusted for sex and age (continuous)

Model 2: adjusted for covariates from Model 1 and additionally adjusted for job group (nursery nurse, nursery nurse assistant, other job group), workplace type (integrated, day care, kindergarten), workplace size (continuous) and baseline scores of outcomes

Model 3: adjusted for covariates from Model 2 and additionally adjusted for intervention status

Model 4: adjusted for covariates from Model 3 and additionally adjusted for the interaction term intervention status × predictor variable

### Supplementary analyses

In the supplementary analyses (see Online Appendix 2), we repeated the analyses shown in Table 2 while using individual-level measures of vertical and horizontal WSC instead of workplace aggregated scores. Results from the supplementary analyses were similar to the results from the main analyses. As in the main analyses, higher levels of vertical and horizontal WSC were associated with a higher level of job satisfaction and a lower level of exhaustion at follow-up in all models. In contrast to the main analyses, a higher level of horizontal WSC was also associated with a lower level of sleep disturbances in the most adjusted model in the supplementary analysis. Vertical WSC showed a suggestive association (*p* = 0.08) with lower levels of sleep disturbances in the most adjusted model in the supplementary analysis.

## Discussion

Using workplace aggregated measures of WSC, we showed that higher levels of two measures of WSC, vertical and horizontal, were both prospectively associated with a higher level of job satisfaction and a lower level of exhaustion in the fully adjusted model in a population of municipal pre-school employees. Further, the analysis of horizontal WSC and sleep disturbances showed a suggestive association. Supplementary analyses repeating the main analyses using individual-level measures of WSC instead of workplace aggregated measures of WSC showed similar results. Previous studies on workplace aggregated WSC and health end points, such as long-term sickness absence (Hansen et al. 2018) and depression (Kouvonen et al. 2008), have shown substantially weaker associations compared to studies using individual-level WSC; however, in our study both workplace aggregated and individual-level WSC strongly predicted higher job satisfaction and lower exhaustion. Only with regard to sleep disturbances, the associations were stronger for individual-level WSC than for workplace aggregated WSC.

To the best of our knowledge, only two studies have previously examined the association between WSC and well-being of employees with a prospective design (Stromgren et al. 2016; Tsuboya et al. 2015). Our findings are in agreement with the findings of these two studies that reported that higher levels of WSC were associated with higher levels of job satisfaction and work engagement (Stromgren et al. 2016) and lower levels of mental distress (Tsuboya et al. 2015). In contrast to our study, the two previous studies used a global measure of WSC and were not able to distinguish between WSC in different relationships. Further, the two previous studies were based on individual-level WSC measures, whereas our study used both workplace aggregated and individual-level measures.

In a recent cross-sectional study on WSC and work engagement, Meng et al. (2018) concluded that WSC may be most appropriately measured at the level of work teams within a workplace, as work teams are the social unit in the workplace that constitutes the most accessible arena for cooperation and social support. In the present study in the pre-school sector, though, workplaces were relatively small and we could with confidence assume that all employees at the workplace were in regular contact with each other. Therefore, we aggregated the individual-level scores not at the work team level, but at the workplace level. As regards larger workplaces where all employees are not in regular contact with each other, WSC may, therefore, most appropriately be measured at the level of work groups.

As delineated in the introduction, WSC may be regarded as a job resource that facilitates the successful execution of work tasks while simultaneously being an important constituent of employees’ well-being (Bakker and Demerouti 2007; Schaufeli and Bakker 2004). Previous studies reported that work-related social resources, such as social support from colleagues (Häusser et al. 2010; Stansfeld and Candy 2006) and supervisors (Nieuwenhuijsen et al. 2010) and quality of leadership (Clausen and Borg 2011; Nielsen and Daniels 2012), were associated with employees’ well-being. Our results are, therefore, in agreement with the findings from these previous studies, with the important addition that the results from our study were based on explanatory variables aggregated to the workplace level.

We used a self-constructed WSC measure because we wanted to tailor the items to the specific working conditions among pre-school teachers in a Danish work environment context. We used six items from the Danish Work Environment Cohort Study (Feveile et al. 2007), one item derived from a questionnaire on relational coordination (Hoffer et al. 2008), and finally two items were developed for the purpose of this study. For example, the item “Are employees involved in decisions regarding workplace changes” was relevant to include because pre-schools in Denmark have been exposed to several changes in the organization of work. Further, a main idea of the study was to study the relation of the psychosocial work environment with conducting work tasks, which is reflected in items like “Our immediate superior contributes to that we can achieve the best possible result” (vertical WSC) and “Is the work distributed fairly” (horizontal WSC).

### Strengths and limitations

The strengths of this study are the prospective design, the use of workplace aggregated measures of WSC and the availability of in total nine WSC items that allowed us to examine two types of WSC, namely vertical and horizontal WSC. An additional strength is that we have information about the workplace (i.e., the unit of aggregation) for all employees both at baseline and at follow-up. The psychometric properties of the two WSC scales were good, with factor analysis clearly showing two distinct factors where all rotated factor loadings were > 0.70 for vertical and > 0.65 for horizontal WSC. Further, both scales had a high internal reliability (Cronbach’s alpha of 0.87 and 0.80 for vertical and horizontal scales, respectively).

There are also weaknesses of the study. We used self-reported data on both exposure and outcome measures, leading to a notable proportion of employees for whom we have only baseline but not follow-up data. Compared to the study population used in the analyses, the population lost to follow-up were statistically significantly younger, more likely to be men, had lower levels of vertical and horizontal WSC and higher exhaustion scores. The size of the dropout proportion may partly be due to the rather long follow-up period of 24 months, which is another weakness of our study, since 2 years can be considered a rather long time lag when analyzing associations between psychosocial exposures and outcomes related to well-being. Ideally, we should have had at least one more measurement point between baseline and follow-up. That would have allowed us to monitor outcomes more closely and it would then have been possible to examine change in WSC (between first and second time point) and the effect of this change on the subsequent outcome (third time point). The lack of repeated measures of WSC will likely have resulted into exposure misclassification and an underestimation of the association between WSC and well-being. Finally, it is a weakness that all three end points have been operationalized from single items in questionnaires, which increases the risk of random measurement errors (Fayers and Machin 2000).

## Conclusion

We conclude that both high vertical workplace social capital, i.e., social capital in the relation between employees and their superiors, and high horizontal workplace social capital, i.e., the social capital in the relation between employees, were prospectively related to better well-being of employees in municipal pre-schools at both the workplace level and the individual level. Workplaces may thus consider focusing on improving workplace social capital as a means for ensuring or improving employees’ well-being.

## Electronic supplementary material

Below is the link to the electronic supplementary material.

Supplementary material 1 (DOCX 24 kb)
