# Supplementary material for: The association of vertical and horizontal workplace social capital with employees’ job satisfaction, exhaustion and sleep disturbances: a prospective study
Source: Int Arch Occup Environ Health. 2019 Apr 9;92(6):883–90. doi: 10.1007/s00420-019-01432-5 (PMC6609764; doi:10.1007/s00420-019-01432-5)
Supplement: Supplementary file 1 — Supplementary material 1 (DOCX 24 kb) [file 420_2019_1432_MOESM1_ESM.docx]

**Appendices for the article: ‘The association of vertical and horizontal workplace social capital with employees’ job satisfaction, exhaustion and sleep disturbances: a prospective study’**

- Appendix 1: Items for measuring vertical and horizontal workplace social capital
- Appendix 2: Associations of individual-level measures of vertical and horizontal workplace social capital with job satisfaction, exhaustion, and sleep disturbances two years later
- Appendix 3: References

**Appendix 1:** Items for measuring vertical and horizontal workplace social capital

**Items for measuring vertical workplace social capital:**

- We have confidence in the management (item derived from the Danish Work Environment Cohort Study (DWECS) 2010 (Det Nationale Forskningscenter for Arbejdsmiljø, 2011))
- The management trusts us to do our work well (item derived from DWECS 2010 (Det Nationale Forskningscenter for Arbejdsmiljø, 2011))
- Our immediate superior contributes to that we can achieve the best possible result (item derived from DWECS 2010 (Det Nationale Forskningscenter for Arbejdsmiljø, 2011))
- Our immediate superior treats us with respect and dignity (item derived from DWECS 2010 (Det Nationale Forskningscenter for Arbejdsmiljø, 2011))
- Are employees involved in decisions regarding workplace changes? (item developed for this study)

Participants were included, if they responded to at least three of the five items on vertical workplace social capital.

**Items for measuring horizontal workplace social capital:**

- We help each other in achieving the best possible result (item derived from DWECS 2010 (Det Nationale Forskningscenter for Arbejdsmiljø, 2011))
- The cooperation between colleagues with different educational backgrounds is good (item developed for this study)
- Do different groups of employees respect each other’s work? (item derived from a questionnaire on relational coordination (Hoffer, Dana, Susan, & Christine, 2008))
- Is the work distributed fairly? (Feveile, Olsen, Burr, & Bach, 2007)

Participants were included, if they responded to at least two of the four items on horizontal workplace social capital.

**Response categories to all nine items:**

1=To a very small extent; 2=To a small extent; 3=Somewhat; 4=To a large extent; 5=To a very large extent

For each of the workplaces we calculated a workplace mean score of the two measures of WSC at baseline and at follow-up. Then, we assigned the workplace mean scores to all individual participants within each of the workplaces. Intra-class correlations were 0.35 and 0.14 for vertical and horizontal WSC, respectively, meaning that 35% and 14% of the variance in the individual level vertical and horizontal WSC, respectively, could be explained by workplace.

**Appendix 2:** Associations of individual-level measures of vertical and horizontal workplace social capital at baseline with job satisfaction, exhaustion, and sleep disturbances two years later

|  | Job satisfaction | | | Exhaustion | | | Sleep disturbances | | |
| --- | --- | --- | --- | --- | --- | --- | --- | --- | --- |
|  | Est | SE | p | Est | SE | p | Est | SE | p |
| Model 1  Vertical social capital | 0.22 | 0.03 | <0.01 | -0.38 | 0.07 | <0.01 | -0.34 | 0.08 | <0.01 |
| Horizontal social capital | 0.25 | 0.05 | <0.01 | -0.42 | 0.08 | <0.01 | -0.41 | 0.10 | <0.01 |
| Model 2  Vertical social capital | 0.17 | 0.04 | <0.01 | -0.20 | 0.08 | 0.01 | -0.19 | 0.08 | 0.01 |
| Horizontal social capital | 0.19 | 0.05 | <0.01 | -0.27 | 0.08 | <0.01 | -0.26 | 0.08 | <0.01 |
| Model 3  Vertical social capital | 0.17 | 0.04 | <0.01 | -0.19 | 0.08 | 0.02 | -0.18 | 0.08 | 0.02 |
| Horizontal social capital | 0.19 | 0.05 | <0.01 | -0.27 | 0.08 | <0.01 | -0.25 | 0.08 | <0.01 |
| Model 4  Vertical social capital | 0.16 | 0.05 | <0.01 | -0.19 | 0.08 | 0.02 | -0.16 | 0.09 | 0.08 |
| Horizontal social capital | 0.18 | 0.05 | <0.01 | -0.26 | 0.09 | <0.01 | -0.23 | 0.09 | 0.01 |

Estimate (Est) and Standard Error (SE) for the association of the baseline score in the predictor variable (vertical and horizontal workplace social capital) with outcomes (job satisfaction, exhaustion, sleep disturbances) two years later. Workplace identification number is included in a repeated statement.

Model 1: Adjusted for sex and age (continuous)

Model 2: Adjusted for covariates from Model 1 and additionally adjusted for job group (nursery nurse, nursery nurse assistant, other job group), workplace type (integrated, day care, kindergarten), workplace size (continuous), and baseline scores of outcomes

Model 3: Adjusted for covariates from Model 2 and additionally adjusted for intervention status

Model 4: Adjusted for covariates from Model 3 and additionally adjusted for the interaction term intervention status*predictor variable

**Appendix 3:** References

Det Nationale Forskningscenter for Arbejdsmiljø. (2011). *Arbejdsmiljø og helbred i Danmark 2010. Resumé og resultater.* Retrieved from: http://www.arbejdsmiljoforskning.dk/~/media/Forside/Arbejdsmiljoedata/Arbejdsmiljo-og-helbred-2010/Samlet-rapport-Arbejdsmiljoe-og-helbred-i-DK-2010.pdf

Feveile, H., Olsen, O., Burr, H., & Bach, E. (2007). Danish Work Environment Cohort Study 2005: From idea to sampling design. *Statistics in Transition, 8*(3), 441-458.

Hoffer, G. J., Dana, W., Susan, P., & Christine, B. (2008). Impact of relational coordination on job satisfaction and quality outcomes: a study of nursing homes. *Human Resource Management Journal, 18*(2), 154-170.
